# Prediction of novel target genes and pathways involved in bevacizumab-resistant colorectal cancer

**DOI:** 10.1371/journal.pone.0189582

**Published:** 2018-01-17

**Authors:** Precious Takondwa Makondi, Chia-Hwa Lee, Chien-Yu Huang, Chi-Ming Chu, Yu-Jia Chang, Po-Li Wei

**Affiliations:** 1 International PhD Program in Medicine, College of Medicine, Taipei Medical University, Taipei, Taiwan; 2 Graduate Institute of Clinical Medicine, College of Medicine, Taipei Medical University, Taipei, Taiwan; 3 School of Medical Laboratory Science and Biotechnology, College of Medical Science and Technology, Taipei Medical University, Taipei, Taiwan; 4 Department of Surgery, College of Medicine, Taipei Medical University, Taipei, Taiwan; 5 Division of General Surgery, Department of Surgery, Shuang Ho Hospital, Taipei Medical University, Taipei, Taiwan; 6 School of Public Health, National Defense Medical Center, Taipei, Taiwan; 7 Cancer Research Center and Translational Laboratory, Department of Medical Research, Taipei Medical University Hospital, Taipei Medical University, Taipei, Taiwan; 8 Division of Colorectal Surgery, Department of Surgery, Wan Fang Hospital, Taipei Medical University, Taipei, Taiwan; 9 Division of Colorectal Surgery, Department of Surgery, Taipei Medical University Hospital, Taipei Medical University, Taipei, Taiwan; 10 Graduate Institute of Cancer Biology and Drug Discovery, Taipei Medical University, Taipei, Taiwan; Columbia University, UNITED STATES

## Abstract

Bevacizumab combined with cytotoxic chemotherapy is the backbone of metastatic colorectal cancer (mCRC) therapy; however, its treatment efficacy is hampered by therapeutic resistance. Therefore, understanding the mechanisms underlying bevacizumab resistance is crucial to increasing the therapeutic efficacy of bevacizumab. The Gene Expression Omnibus (GEO) database (dataset, GSE86525) was used to identify the key genes and pathways involved in bevacizumab-resistant mCRC. The GEO2R web tool was used to identify differentially expressed genes (DEGs). Functional and pathway enrichment analyses of the DEGs were performed using the Database for Annotation, Visualization, and Integrated Discovery(DAVID). Protein–protein interaction (PPI) networks were established using the Search Tool for the Retrieval of Interacting Genes/Proteins database(STRING) and visualized using Cytoscape software. A total of 124 DEGs were obtained, 57 of which upregulated and 67 were downregulated. PPI network analysis showed that seven upregulated genes and nine downregulated genes exhibited high PPI degrees. In the functional enrichment, the DEGs were mainly enriched in negative regulation of phosphate metabolic process and positive regulation of cell cycle process gene ontologies (GOs); the enriched pathways were the phosphoinositide 3-kinase-serine/threonine kinase signaling pathway, bladder cancer, and microRNAs in cancer. Cyclin-dependent kinase inhibitor 1A(*CDKN1A*), toll-like receptor 4 (*TLR4*), CD19 molecule (*CD19*), breast cancer 1, early onset (*BRCA1*), platelet-derived growth factor subunit A (*PDGFA*), and matrix metallopeptidase 1 (*MMP1*) were the DEGs involved in the pathways and the PPIs. The clinical validation of the DEGs in mCRC (TNM clinical stages 3 and 4) revealed that high PDGFA expression levels were associated with poor overall survival, whereas high BRCA1 and MMP1 expression levels were associated with favorable progress free survival(PFS). The identified genes and pathways can be potential targets and predictors of therapeutic resistance and prognosis in bevacizumab-treated patients with mCRC.

## Introduction

Colorectal cancer (CRC) is the third most frequently diagnosed cancer and the second leading cause of cancer deaths worldwide, accounting for 10% of the worldwide cancer incidence and mortality [1]. Surgery is the treatment of choice for nonmetastatic CRC; however, approximately 20% of cases present with metastatic disease at the time of diagnosis and half of the patients experience recurrence and metastases even after complete resection of the primary tumor, leading to a poor prognosis and median overall survival (OS) of approximately 24 months [2, 3]. The inclusion of cytotoxic agents (irinotecan and oxaliplatin) in fluoropyrimidine (intravenous 5-fluorouracil or oral capecitabine)-based systemic chemotherapy has been reported to improve the associated response rates (RR) from 15%–20% to 30%–40%, time to progression from 5–6 to 8 months, and OS from 10–12 to 20–24 months [3–7]. Furthermore, therapeutic benefits have been demonstrated to increase through the use of targeted drugs, such as angiogenesis inhibitors (bevacizumab, ziv-aflibercept, and ramucirumab) and antiepidermal growth factor receptor antibodies (cetuximab and panitumumab), as the first and second lines of treatment in patients with with K-RAS-wild-type tumors tumors [8–12].

Bevacizumab is the first agent to influence OS in patients with metastatic CRC (mCRC); when combined with irinotecan-based chemotherapy, the median OS improved from 15.6 to 20.3 months, median PFS from 6.2 to 10.6 months and RR from 34.8% to 44.8%[10]. The addition of bevacizumab to oxaliplatin-based chemotherapy improved median PFS from 8.0 to 9.4 months though there was no significant difference in OS(19.9 to 21.3 months) [13], while in previously treated mCRC; oxaliplatin based therapy improved both OS and PFS (10.8 to 12.9 months and 4.7 to 7.3 respectively)[14]. When compared for effectiveness, the irinotecan based chemotherapy has shown to have an edge over oxaliplatin based chemotherapy with the addition of bevacizumab (OS = 31.4 vs 30.1 months, PFS = 12.1 vs 10.7 months)[15]. These results have also been echoed in the MAVERICC trial (OS = 27.5 vs 23.9 months, PFS = 12.6 vs 10.1 months)[16]. Bevacizumab is a humanized monoclonal antibody that binds to vascular endothelial growth factor A (VEGF-A) and thus prevents interaction with its receptors, VEGFR-1 (Flt-1) and VEGFR-2 (Flk-1/KDR), leading to the regression of existing tumor blood vessels, normalization of the remaining blood vessels, and consequently tumor inhibition [17]. However, the therapeutic effects of bevacizumab are strongly affected by the lack of biomarkers that can facilitate selecting a population that might benefit from this medication and can predict therapeutic resistance [18–20].

In this study, we investigated the predictive biomarkers and pathways of bevacizumab resistance in mCRC by using microarray data from the Genetic Expression Omnibus (GEO) database. The new biomarkers were assessed for their ability to predict OS and PFS. The identification of predictive and prognostic biomarkers can facilitate improving the therapeutic index of bevacizumab.

## Materials and methods

### Microarray data

The gene expression profile of GSE86525 was obtained from the GEO (http://www.ncbi.nlm.nih.gov/geo/) database [21], which was sequenced on the GPL16699 platform of Agilent-039494 SurePrint G3 Human GE v2 8 × 60K Microarray 039381 (Agilent Technologies, Santa Clara, CA, USA). The GSE86525 dataset includes microarray gene expression data derived from three bevacizumab-resistant HT29 xenograft tumors and three untreated HT29 xenograft tumors as controls. In brief, HT29 cells (1 × 10^7^) suspended in phosphate-buffered saline were subcutaneously injected into the flanks of BALB/c nude mice, and the tumor-bearing mice were treated with bevacizumab (5 mg/kg, twice a week) for 3 weeks to obtain bevacizumab-resistant tumors. MTT colorimetric assays were used to determine the 50% inhibitory concentration for bevacizumab-resistant and untreated xenograft tumors; the tumor sizes were compared between the two groups. The sample tissues were immediately frozen under liquid nitrogen after isolation. Total RNAs were extracted from the samples, evaluated, labeled and hybridized, using a SurePrint G3 Human GE 8 × 60K microarray (Agilent Technologies). Array images were captured using a DNA microarray scanner (Agilent Technologies), and the data were analyzed using Feature Extraction Software (Agilent Technologies) to obtain background-corrected signal intensities. The expression data were further analyzed using GeneSpring GX software (version 11.0, Agilent Technologies), and the differentially expressed genes (DEGs) between the bevacizumab-resistant HT29 tumors vs untreated control were compared using the Fisher exact test, followed by multiple corrections using the Benjamini and Hochberg false discovery rate (FDR) method [22]. Gene sets with an FDR q-value of <0.05 were considered statistically significant, and all experiments were performed in triplicate.

### Data preprocessing and DEGs screening

The data were recalculated using the GEO2R analytical tool to identify the DEGs associated with acquired bevacizumab-resistant CRC [23, 24]. The *t* test and Benjamini and Hochberg method were used to calculate the P values and FDR, respectively [22]. The genes were considered to be differentially expressed for an FDR value of <0.05 and fold change (FC) of >2 or <-2 (log2FC > 1 or < -1). The DEG expression data were extracted, and a bidirectional hierarchical clustering plot was constructed using MultiExperiment Viewer (MeV; version 4.8) software [25].

### Construction of PPI networks

Protein–protein interaction (PPI) networks were plotted using the Search Tool for the Retrieval of Interacting Genes/Proteins (STRING; version 10.0; http://www.string-db.org/), an online database comprising comprehensive known and predicted interactions, to determine the interactive relationships among the DEG-encoded proteins. A combined score of >0.7 (high confidence) was used as the cutoff criterion [26]. PPI pairs were visualized using Cytoscape software (version 3.4.0; http://www.cytoscape.org/), and the CytoNCA tool was used to subcluster the plotted PPI networks [27–30]. Highly connected proteins with important biological functions were identified by calculating the degree (number of line connections between proteins) and the betweenness value (fraction of the number of shortest paths that pass through each node; A measure of how often nodes occur on the shortest paths between other nodes) of each node with a degree cutoff criterion of ≥2.

### Enrichment analysis of DEGs

The Database for Annotation, Visualization, and Integrated Discovery (DAVID, http://david.abcc.ncifcrf.gov/) was used to classify the DEGs involved in the PPI networks according to their biological processes, molecular functions, or cellular components by using the Gene Ontology (GO) Consortium Reference (http://www.geneontology.org/) [31, 32]. Gene sets with a P value of <0.05 and FDR value of <0.05 were considered statistically significant. In addition, the DAVID tool was used for pathway enrichment analysis, and the reference pathways were obtained from the Kyoto Encyclopedia of Genes and Genomes (KEGG; http://www.genome.jp/kegg/) database website to perform KEGG pathway enrichment analysis for the DEGs involved in the PPI networks, with a P value of <0.05 and FDR value of <0.05 being considered statistically significant [33, 34].

### Clinical validation of the DEGs

The clinical assessment of DEGs associated with bevacizumab resistance was performed using the SurvExpress tool [35]. The colon metabase, which includes GSE12945[36], GSE14333[37], GSE17536[38], GSE17537[38], GSE31595, and GSE41258[39] with a total of 808 cases, was used in this study. Survival profiles were compared on the basis of a high or low mRNA expression level of a particular gene, and they were censored independently for OS and PFS in months and stratified further according to TNM clinical stages 3 and 4. A log-rank P value of <0.05 was considered statistically significant, and the data were analyzed using SPSS for Macintosh (version 21, IBM Corp Armonk, NY, USA; www-01.ibm.com) for plotting Kaplan–Meier survival curves.

### Gene co-expression in colorectal cancer data

The Cancer Genome Atlas (TCGA; https://cancergenome.nih.gov/) was used to obtain CRC data containing gene expression profiles. Level 3 RNASeq data containing gene expression profiles of 635 CRC cases (colon adenocarcinoma, N = 463; and rectal adenocarcinoma, N = 172) were obtained. The standard Pearson correlation coefficients (-1 to 1) and the coefficient of variation (the ratio of standard deviation to mean) of the desired gene pairs were calculated using SPSS for Macintosh (version 21, IBM Corp., Armonk, NY, USA; https://www-01.ibm.com). A P value of <0.05 was considered statistically significant and was used as the cutoff criterion.

## Results

### DEGs screening and heat map clustering analysis

**Fig 1:**
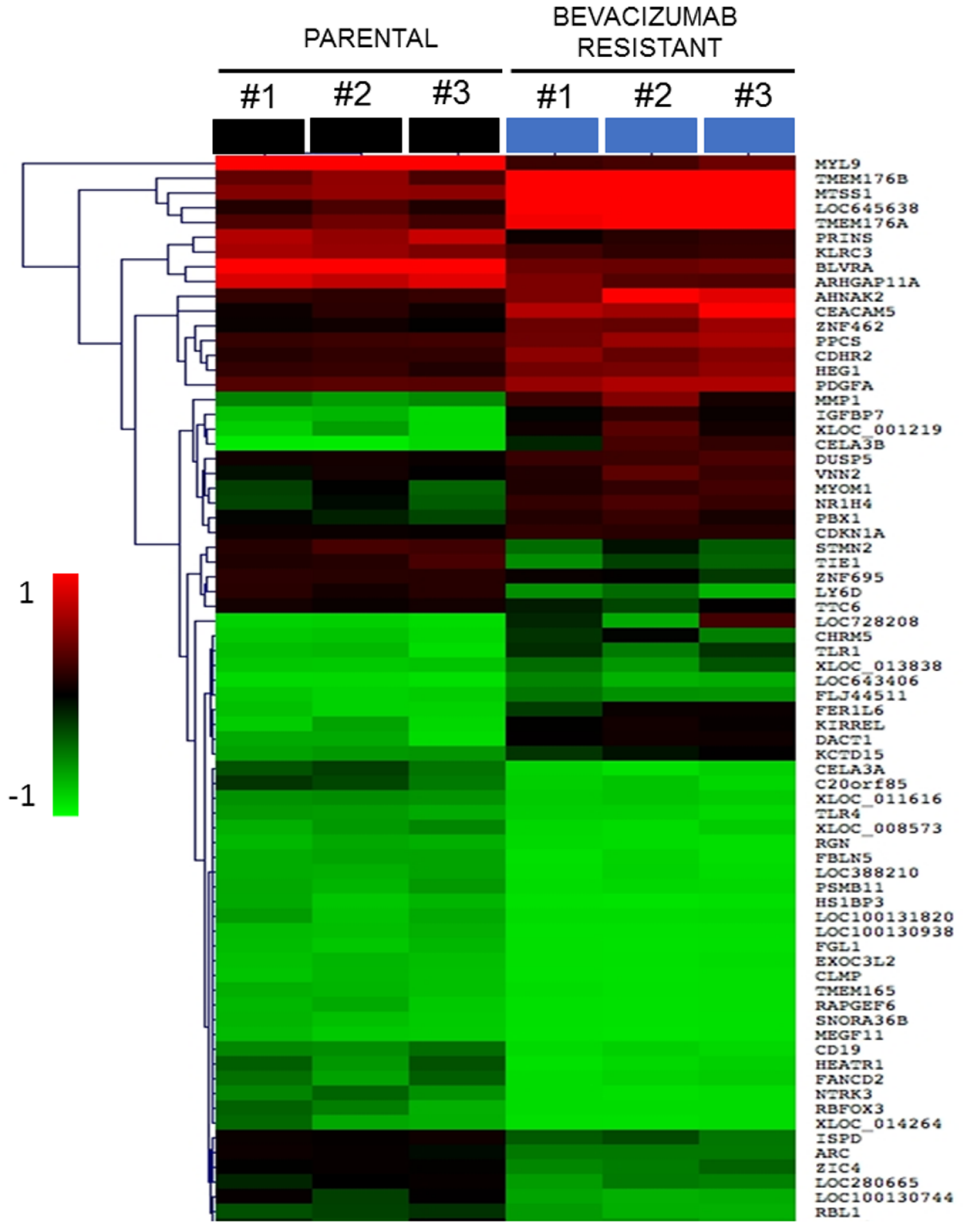
Heat map showing up-regulated and down-regulated differentially expressed genes (DEGs) in bevacizumab-resistant colon cancer tumors. A bidirectional hierarchical clustering heat map was constructed using MultiExperimental Viewer(MeV). The expression values are log2 fold changes (>1 or <−1, FDR <0.05)) between corresponding bevacizumab -resistant HT29 xenograft tumors and non-treated HT29 xenograft tumors. Black represents no change in expression, green represents down-regulation, and red represents up-regulation.

The GEO2R tool was used to identify DEGs from the data derived from the GPL16699 oligonucleotide microarray platform, comprising 62,976 probe sets. A total of 124 DEGs were determined to be associated with bevacizumab resistance, with 57 being upregulated and 67 being downregulated, as determined according to their log2FC and FDR values (S1 and S2 Tables). MeV software was used to construct a heat map to obtain the bidirectional hierarchical clustering of the DEGs and summarize the upregulated and downregulated DEGs (Fig 1).

### PPI network analysis

**Fig 2:**
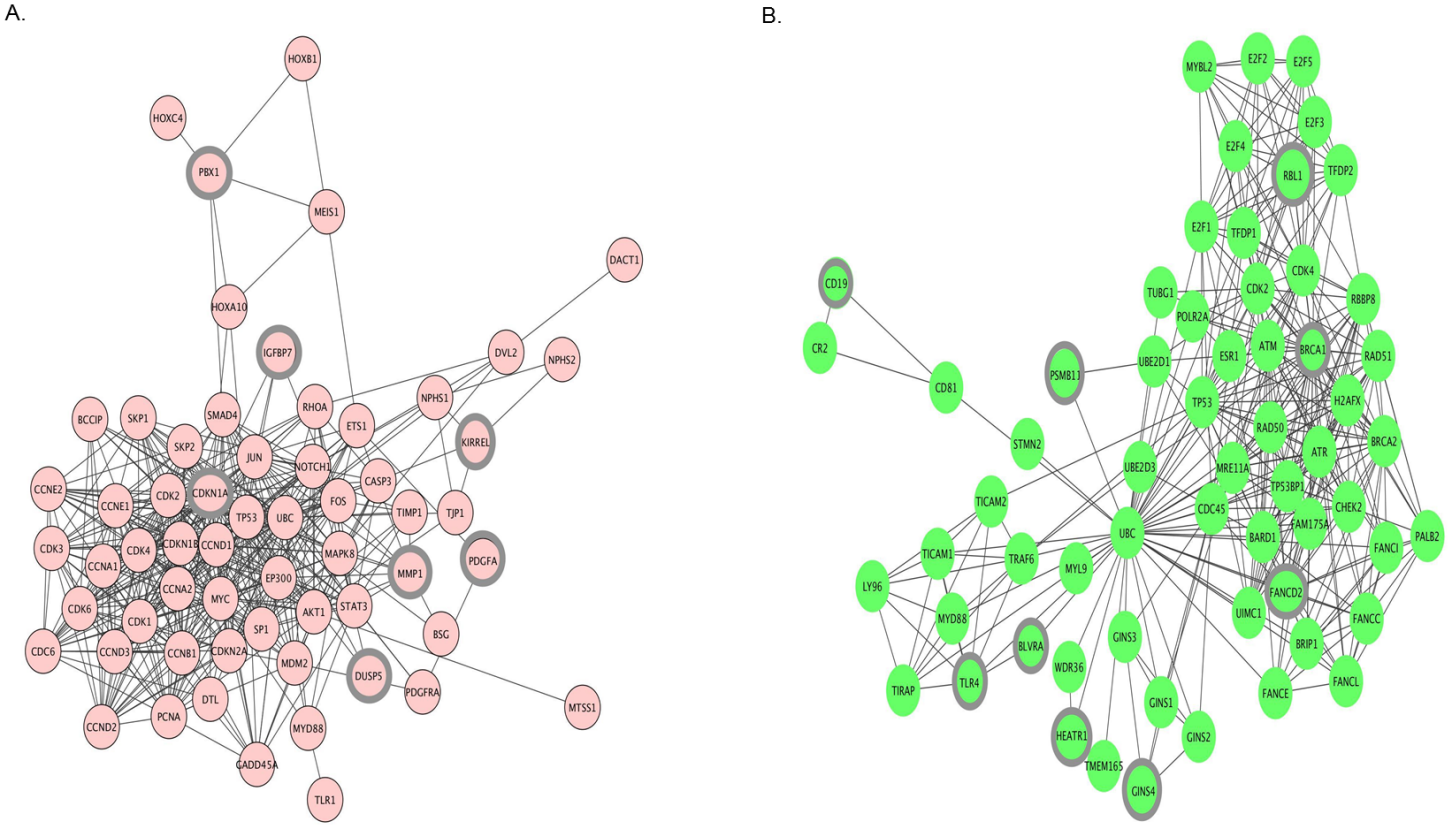
Protein–protein interaction (PPI) network of differentially expressed genes(A) up-regulated genes and (B) down-regulated genes. The PPI pairs were imported into Cytoscape software as described in methods and materials. Pink nodes represent up-regulated genes while green nodes represent down-regulated genes. The lines represent interaction relationship between nodes. The highlighted DEGs represents degree = >2.

The PPI pairs obtained using the STRING database were visualized using Cytoscape software and analyzed using the CytoNCA plugin. The upregulated network had 88 nodes and 466 edges. Seven genes, namely cyclin-dependent kinase inhibitor 1A (CDKN1A; p21 and Cip1), matrix metallopeptidase 1 (MMP1; interstitial collagenase), pre-B cell leukemia transcription factor 1 (PBX1), platelet-derived growth factor alpha polypeptide (PDGFA), kin of IRRE-like (Drosophila) (KIRREL), insulin-like growth factor binding protein 7 (IGFBP7), and dual specificity phosphatase 5 (DUSP5), exhibited higher PPI degrees and betweenness values (Fig 2A and Table 1). In the downregulated network, containing 88 nodes and 350 edges, nine genes, namely breast cancer 1, early onset (BRCA1), retinoblastoma-like 1 (p107) (RBL1), toll-like receptor 4 (TLR4), CD19, HEAT repeat-containing 1 (CD19), Fanconi anemia complementation group D2 (FANCD2), proteasome subunit beta 11 (PSMB11), biliverdin reductase A (BLVRA), and GINS complex subunit 4 (GINS4), showed higher PPI degrees and betweenness values (Fig 2B, Table 2).

**Table 1:** Up-regulated genes which had interactions in the PPIs.

Gene symbol	Gene name	Degree	Betweenness
CDKN1A	cyclin-dependent kinase inhibitor 1A (p21, Cip1)	38.0	209.46272
MMP1	matrix metallopeptidase 1 (interstitial collagenase)	10.0	20.48990
PBX1	pre-B-cell leukemia homeobox 1	5.0	192.13673
KIRREL	kin of IRRE like (Drosophila)	4.0	46.28517
IGFBP7	insulin-like growth factor binding protein 7	4.0	0.44311
DUSP5	dual specificity phosphatase 5	3.0	0.44311
PDGFA	platelet-derived growth factor alpha polypeptide	2.0	2.66667

**Table 2:** Down-regulated genes which had interactions in the PPIs.

Gene symbol	Gene name	Degree	Betweenness
BRCA1	breast cancer 1, early onset	35.0	326.96054
FANCD2	fanconi anemia complementation group D2	19.0	33.54729
RBL1	retinoblastoma-like 1 (p107)	13.0	12.75956
TLR4	toll-like receptor 4	8.0	31.75171
GINS4	GINS complex subunit 4	4.0	0.0
CD19	CD19 molecule	2.0	0.0
HEATR1	HEAT repeat containing 1	2.0	0.0
PSMB11	proteasome subunit beta 11	2.0	0.0
BLVRA	biliverdin reductase A	2.0	0.0

### Functional enrichment analysis

The DAVID tool was used to classify the DEGs involved in the PPI networks according to their common biological processes, molecular functions, or cellular components. Of the 1,454 GO gene sets included from the reference database, 111 were significantly enriched (P < 0.05; FDR < 0.05). Table 3 lists the top five gene sets, with those involved in the negative regulation of phosphate metabolic process and positive regulation of cell cycle process being the most significant and they include DUSP5, CDKN1A (p21 and Cip1), KIRREL, PDGFA, TLR4, PSMB11, BRCA1, and PBX1.

**Table 3:** Enriched Gene-Ontologies (GO’s).

Gene-Ontology	Genes	p-value	FDR^a^
negative regulation of phosphate metabolic process (GO:0045936)	DUSP5, CDKN1A, KIRREL, PDGFA, TLR4	0.00002184	0.004597
positive regulation of cell cycle process (GO:0090068)	PSMB11, CDKN1A, BRCA1, PBX1	0.00002337	0.004597
regulation of lipid metabolic process (GO:0019216)	RBL1, PDGFA, IGFBP7, BRCA1	0.00003562	0.004597
positive regulation of cell cycle arrest (GO:0071158)	PSMB11, CDKN1A, BRCA1	0.00003986	0.004597
DNA damage response, signal transduction by p53 class mediator (GO:0030330)	PSMB11, CDKN1A, BRCA1	0.00004889	0.004597

As there were 111 enriched Gene-Ontologies, here we only present top 5 most significant terms according to P-value and

^a^FDR (False discovery rate).

GO: gene-ontology

### KEGG pathway analysis

**Fig 3:**
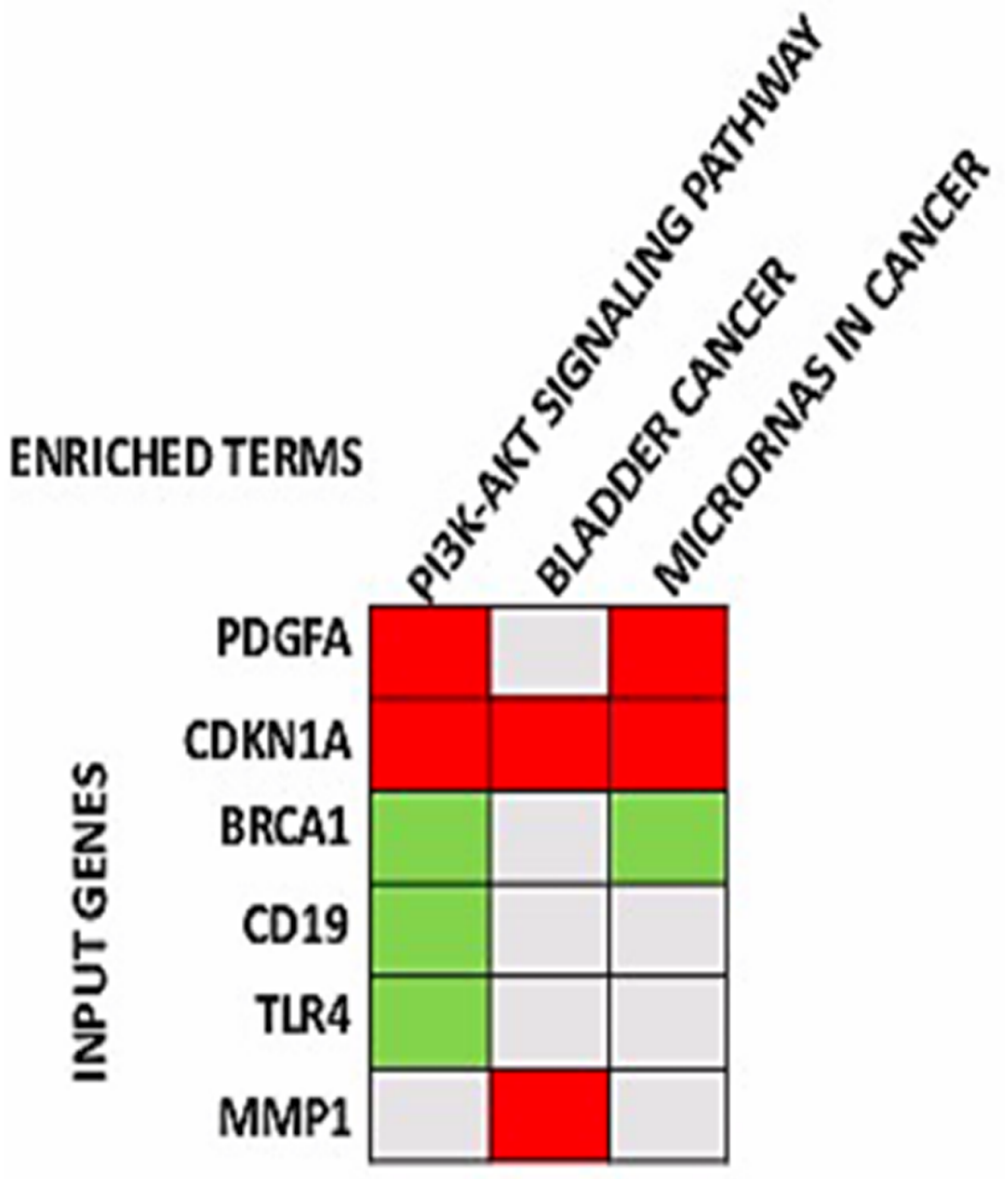
Significant KEGG pathways and the genes involved. Gene enrichment analysis showing KEGG pathways significantly enriched in bevacizumab resistant HT29 xenograft tumors and the genes involved in the pathways (the pathways are in order of their enrichment from left to right) (FDR <0.05, p-value <0.05).

The DAVID tool was applied to classify the DEGs involved in the PPI networks by using the reference pathways from KEGG. KEGG pathway analysis revealed significant results (P < 0.05; FDR < 0.05) for three pathways: the phosphoinositide 3-kinase-serine/threonine kinase (PI3K-AKT) signaling pathway (involving CD19, BRCA1, PDGFA, CDKN1A, and TLR4), bladder cancer (involving CDKN1A and MMP1), and microRNAs in cancer (involving CDKN1A, PDGFA, and BRCA1; (Fig 3, Table 4)

**Table 4:** Enriched KEGG pathways.

KEGG^a^ pathway	Genes	p-value	FDR^b^
PI3K-Akt signaling pathway (hsa04151)	CDKN1A, CD19, PDGFA, BRCA1, TLR4	0.000005237	0.0003037
Bladder cancer (hsa05219)	CDKN1A, MMP1	0.000483151	0.009340926
MicroRNAs in cancer (hsa05206)	CDKN1A, PDGFA, BRCA1	0.001572901	0.01303

^a^KEGG: Kyoto Encyclopedia of Genes and Genome

^b^FDR:False discovery rate

### Survival analysis of the enriched DEGs

**Fig 4:**
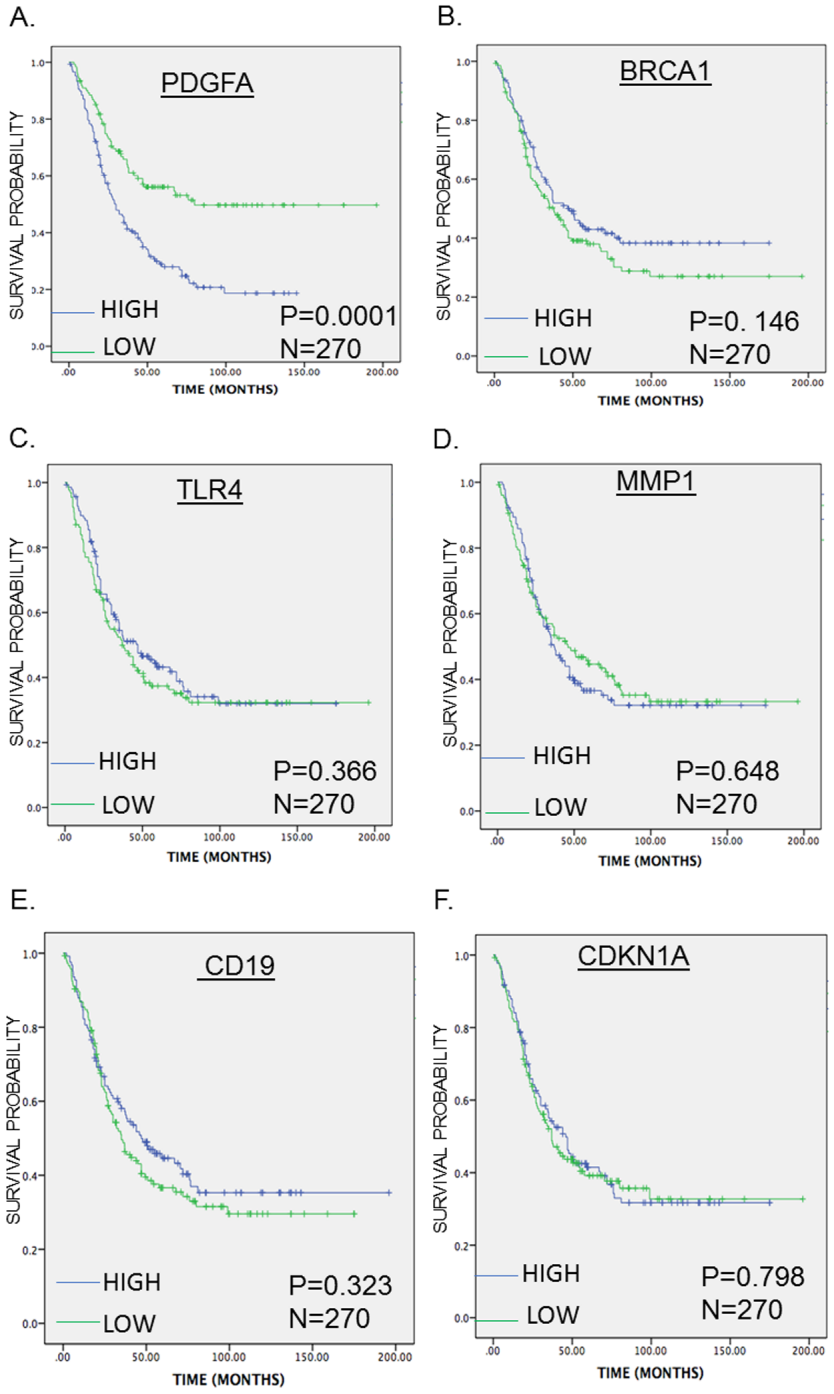
Kaplan-Meier survival curves presenting the prognostic relationship between high and low expression of specific genes involved in bevacizumab resistance to overall survival (OS) in TNM clinical stage 3 and 4(A) PDGFA, (B) BRCA1, (C) TLR4, (D) MMP1 (E) CD19 and (F) CDKN1A expression. The survival curves were plotted using the *survExpress* online tool. The specific DEGs expression levels were dichotomized by median value and stratified for TNM clinical stage. The results presented visually by Kaplan-Meier survival plots. p-values were calculated using log-rank statistics. Patient number(N) = 270, p = Logrank p-value.

**Fig 5:**
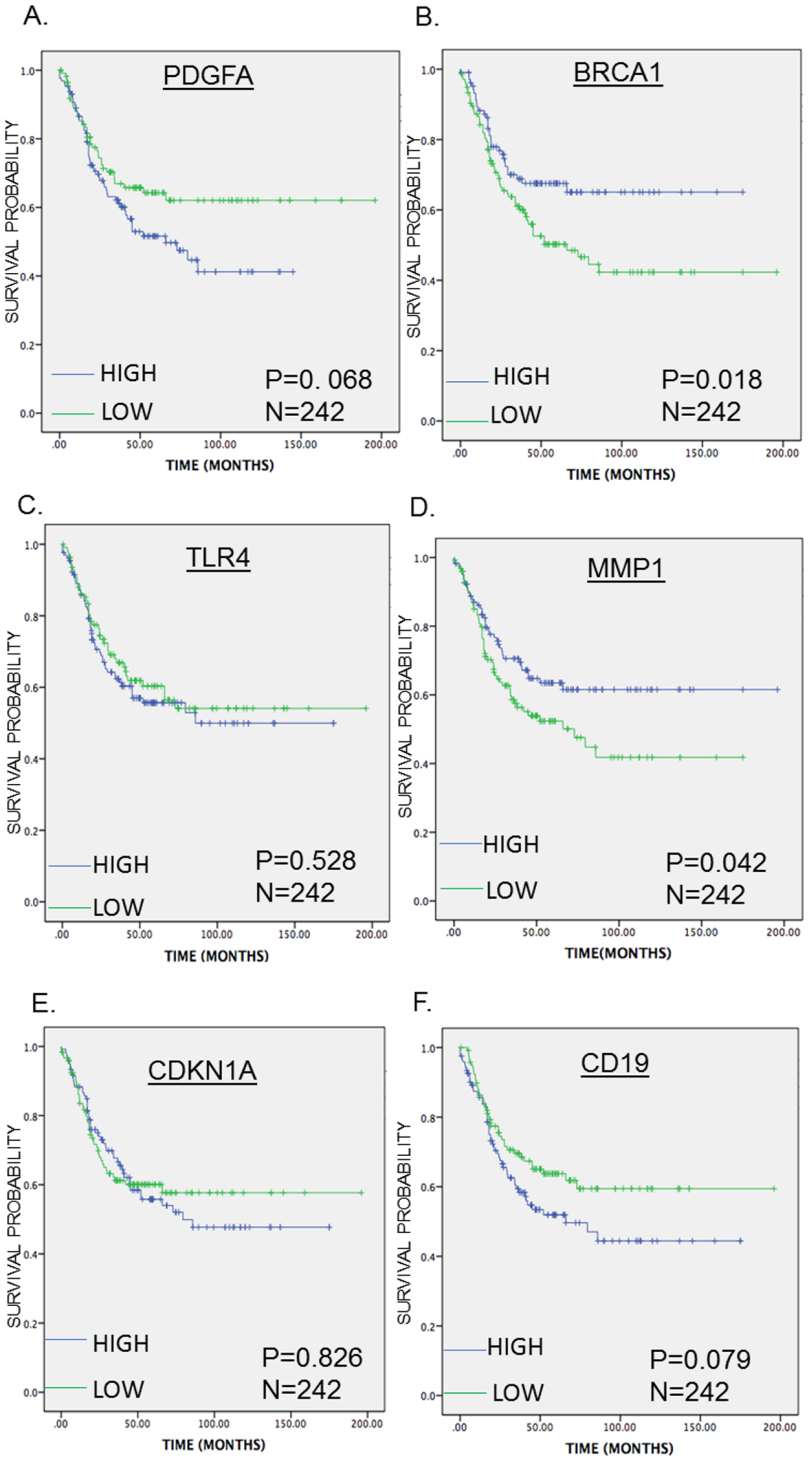
Kaplan-Meier survival curves presenting the relationship between high and low expression of specific genes involved in bevacizumab resistance to Progress Free survival in TNM clinical stage 3 and 4 (A) PDGFA, (B) BRCA1, (C) TLR4, (D) MMP1 (E) CDKN1A and (F) CD19 expression. The survival curves were plotted using the *survExpress* online tool. The specific DEGs expression levels were dichotomized by median value and stratified for TNM clinical stage. The results presented visually by Kaplan-Meier survival plots. P-values were calculated using log-rank statistics. Patient number(N) = 242, p = Logrank p-value.

The SurvExpress tool was used to assess the enriched DEGs for their ability to predict OS and PFS in mCRC. High PDGFA expression levels were associated with poor OS, whereas high BRCA1 and MMP1 expression levels were associated with favorable PFS. However, the expression levels of CD19, CDKN1A, and TLR4 were neither associated with OS nor PFS (Figs 4 and 5).

### Mechanism of gene correlation in tumor tissues

**Fig 6:**
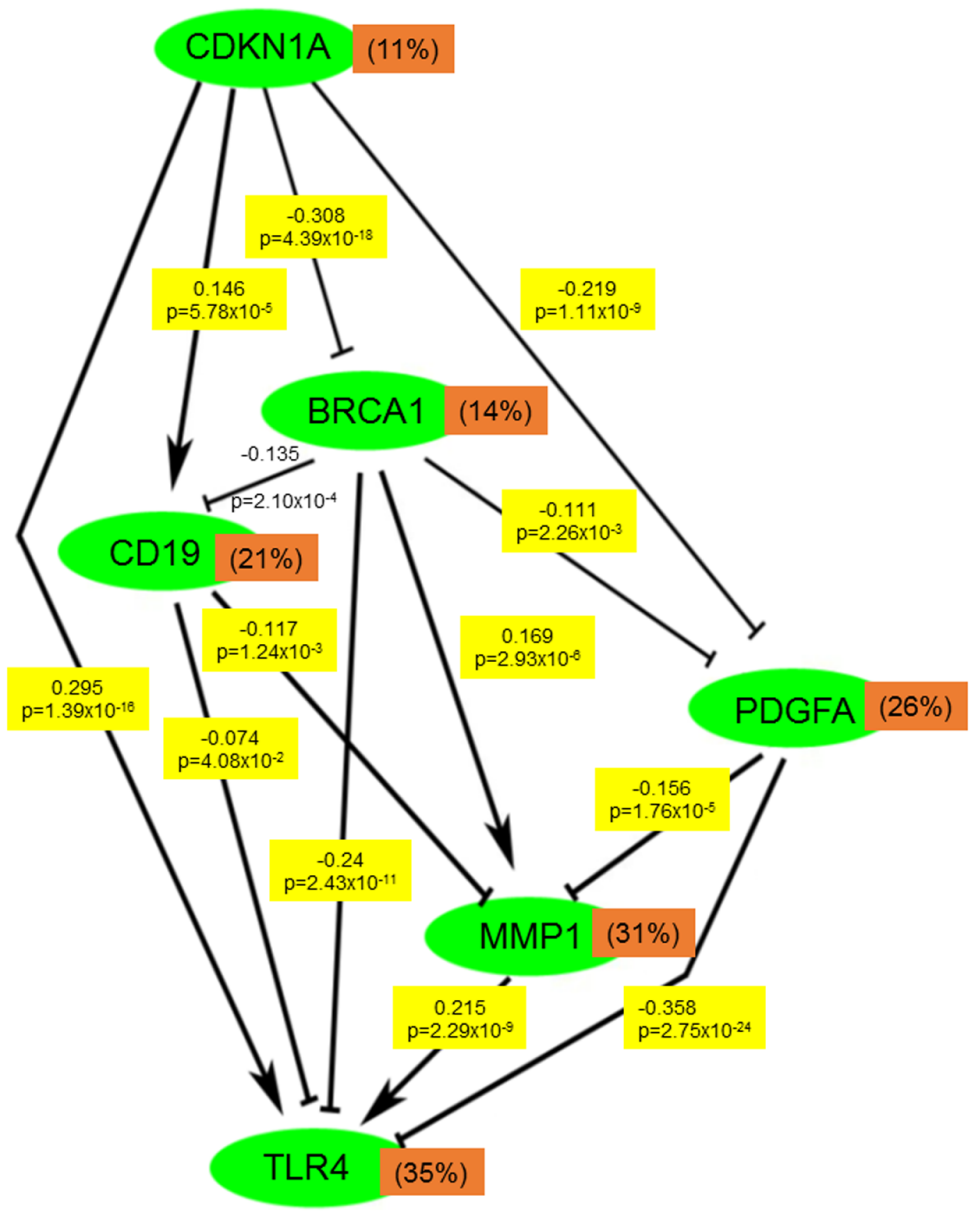
Gene expression correlation of the DEGs involved in the pathways in the colorectal carcinoma tumor samples from TCGA data base. TCGA RNASeq Level 3 data was used and Pearsons correlation coefficients (-1 to 1) were calculated. Number of patients = 653. The percentage presents the coefficient of variation of the genes, the lines presents negative correlation and the arrow presents positive correlation (p-value <0.05).

To elucidate the mechanism underlying the gene–gene correlation of the DEGs, TCGA RNASeq level 3 CRC data were used. BRCA1 was negatively correlated with PDGFA, CDKNA1, CD19, and TLR4 and positively correlated with MMP1. Moreover, PDGFA was negatively correlated with CDKNA1, BRCA1, MMP1, and TLR4. TLR4 was positively correlated with CDKNA1 and MMP1 and negatively correlated with CD19 and BRCA1. Furthermore, CD19 was positively correlated with CDKNA1 and negatively correlated with BRCA1, MMP1, and TLR4. However, PDGFA and CD19 were not significantly correlated (Fig 6).

## Discussion

The overall mortality of CRC has remained unchanged over the past decades, despite advances in surgical and medical therapy [40, 41]. This is due to the difficulties associated with early detection of the disease and the development of acquired therapeutic resistance, leading to ineffective treatment in patients with metastatic diseases [42–44]. Therefore, the etiological factors and mechanisms of acquired therapeutic resistance must be explored to improve survival rates and prevent disease recurrence [43]. Microarray technology has been widely used in the identification of therapeutic targets for diagnosis and prognosis of cancers [45, 46]. Our study performed a systematic bioinformatic analysis of the microarray data of HT29 xenograft tumor models with acquired bevacizumab resistance and identified 124 DEGs, 57 of which were upregulated and 67 were downregulated. CD19, BRCA1, PDGFA, CDKNA1, MMP1, and TLR4 exhibited high PPI degrees and were enriched in the PI3K-AKT signaling pathway, bladder cancer, and microRNAs in cancer; however, only high PDGFA expression levels were associated with poor OS, whereas high BRCA1 and MMP1 expression levels were associated with favorable PFS. These discrepancies may be because the study cohort was not specifically on bevacizumab treatment, thus suggesting that biomarkers that predict OS do not specifically predict PFS. Therefore, to confidently interpret the study results, these biomarkers require further assessment in patients specifically treated with bevacizumab.

The results of this study reveal PDGFA overexpression to be associated with bevacizumab resistance and the prognosis of patients with mCRC. These results are consistent with those of a previous study, which identified PDGFA as a potential predictor of therapeutic resistance and an individual prognostic marker for bevacizumab treatment, because PDGFA expression was observed to be decreased after single-dose bevacizumab treatment in responders but remained unchanged in nonresponders [47]. PDGFA targeting with the PDGF receptor has been reported to increase chemotherapeutic sensitivity in different cancers [47–50]. Therefore, our study supports the current understanding that PDGFA acts not only as a predictor of treatment response but also as a prognostic factor, because PDGFA upregulation not only limited the response to bevacizumab but also affected the prognosis of patients with mCRC in this study. Notably, PDGF overexpression has been implicated in bevacizumab resistance and poor prognosis in bevacizumab-treated patients because the PDGF pathway is considered an alternative pathway in the development of bevacizumab resistance [51, 52].

The expression levels of MMP1 and BRCA1 were associated with PFS in patients with mCRC. Although this study is the first to demonstrate the aforementioned relationship in mCRC, MMPs have received attention in terms of their role in the mechanism underlying resistance to antiangiogenic therapy, because increased MMP2 and MMP9 expression levels have been associated with resistance to the anti-VEGF and antiplacental growth factor drug aflibercept and with poor OS [53, 54]. Furthermore, MMP1 expression has been strongly associated with tumor metastasis and adverse outcomes in mCRC and has been suggested as a potential prognostic and therapeutic target [55–59]. A previous study reported that BRCA1 is associated with early onset CRC and functions as a DNA repair gene to cytotoxic drugs [60]. BRCA1 has been considered as a predictor of treatment response and prognosis in breast, ovarian, and lung cancers [61–66]; however, its role in mCRC and bevacizumab resistance is yet to be explored. The present results suggest that BRCA1 may exert protective effects in mCRC; therefore, BRCA1 should be thoroughly studied because BRCA1 targeting might not only increase the prognostic and therapeutic effects of bevacizumab but also affect the expression levels of its associated genes, namely PDGFA, CDKN1A, TLR4, and MMP1.

CD19, CDKN1A, and TLR4 have also been reported to influence therapeutic resistance or overall prognosis in cancer. CD19 has been associated with chemotherapy and multidrug resistance in many hematological tumors, and plays a central role in targeted therapeutics against B-cell malignancies (because of its expression patterns throughout the B-cell lineage), and against most B-cell malignancies with successful preclinical experiments and first-generation clinical trials [67–72]. CDKN1A has been implicated in cell cycle regulation, cell death, DNA repair, and cell motility [73]. Studies have demonstrated CDKN1A overexpression to be associated with poor prognosis in gastric and esophageal carcinomas [74, 75]. Furthermore, studies have reported that TLR4 plays a role in CRC; polymorphisms increasing TLR4 signaling led to a highly aggressive CRC, whereas those reducing TLR4 signaling exerted protective effects [76, 77]. In addition, high TLR4 expression levels have been associated with highly advanced grades of colonic neoplasia and with lower OS, a high probability of CRC relapse, and the presence of liver metastases in humans [78–81]. Studies have also suggested TLR4 to promote angiogenesis in different cancers by activating the PI3K-AKT signaling pathway to induce VEGF expression. In addition, TLR4 inhibition is associated with VEGF inhibition [82–84]. This finding can explain TLR4 downregulation in the bevacizumab-resistant tumors in this study; however, in vitro validation of this finding is required.

Notably, five of the six genes that were commonly enriched as well as associated with bevacizumab resistance belonged to the PI3K-AKT signaling pathway. Therefore, we suggest that the PI3K-AKT signaling pathway is responsible for restraining the therapeutic efficacy of bevacizumab in mCRC. This observation is in accordance with the results of previous studies, which have suggested that modifications in the PI3K-AKT signaling pathway increase bevacizumab resistance as an alternative pathway to VEGF inhibition [85–87]. Moreover, the occurrence of mutations in the PI3K-AKT signaling pathway remains the main challenge for mCRC treatment with new biological agents [86, 88, 89].

The present findings provide novel data that could predict bevacizumab treatment response and the emergence of resistance. Furthermore, this approach can predict patient prognosis; however, additional studies are required to validate the study findings and determine their clinical applicability.

## Supporting information

S1 Table Down-regulated bevacizumab-resistant genes. (DOCX)

S2 Table Up-regulated genes. (DOCX)
