# Supplementary material for: Prediction of novel target genes and pathways involved in bevacizumab-resistant colorectal cancer
Source: PLoS One. 2018 Jan 17;13(1):e0189582. doi: 10.1371/journal.pone.0189582 (PMC5771567; doi:10.1371/journal.pone.0189582)
Supplement: S1 Table — (DOCX) [file pone.0189582.s001.docx]

**S1 Table: Down-regulated genes**

| **GENE SYMBOL** | **LOG2FC** | **P-VALUE** |
| --- | --- | --- |
| LY6D | -2.317 | 0.0003583 |
| MYL9 | -2.195 | 0.0003571 |
| TIE1 | -2.093 | 0.0006425 |
| CELA3A | -2.074 | 0.0001572 |
| STMN2 | -2.041 | 0.000328 |
| C17orf13 | -2.018 | 0.0000686 |
| HEATR1 | -1.959 | 0.0006046 |
| NTRK3 | -1.929 | 0.0000347 |
| C20orf85 | -1.826 | 0.0003584 |
| XLOC l2 014802 | -1.815 | 0.0011117 |
| PRINS | -1.768 | 0.0011122 |
| RBFOX3 | -1.765 | 0.0009639 |
| FAM176A | -1.741 | 0.0001745 |
| XLOC 014264 | -1.658 | 0.0011847 |
| CD19 | -1.651 | 0.0000797 |
| FANCD2 | -1.62 | 0.0011447 |
| FAM176A | -1.582 | 0.0002792 |
| LOC100130744 | -1.58 | 0.0004305 |
| BRCA1 | -1.554 | 0.0005905 |
| LOC100130579 | -1.47 | 0.0014378 |
| XLOC l2 013808 | -1.418 | 0.0001888 |
| CELA3A | -1.341 | 0.0009624 |
| HS1BP3 | -1.34 | 0.0004476 |
| FBLN5 | -1.339 | 0.0002495 |
| XLOC l2 001760 | -1.334 | 0.0009679 |
| LOC100507624 | -1.306 | 0.0001878 |
| SNAR-F | -1.303 | 0.0005897 |
| SNAR-G2 | -1.3 | 0.0005357 |
| SNAR-H | -1.294 | 0.0004036 |
| XLOC l2 013462 | -1.279 | 0.0002804 |
| XLOC 008573 | -1.256 | 0.0014016 |
| XLOC 000884 | -1.251 | 0.0009284 |
| PSMB11 | -1.212 | 0.0005414 |
| LOC388210 | -1.206 | 0.0005505 |
| ZNF695 | -1.202 | 0.0013288 |
| RAPGEF6 | -1.198 | 0.001134 |
| LOC729178 | -1.186 | 0.0003242 |
| LOC100131820 | -1.179 | 0.0013815 |
| LOC100130938 | -1.173 | 0.0001377 |
| RGN | -1.172 | 0.0001823 |
| TMEM165 | -1.161 | 0.0002761 |
| BLVRA | -1.157 | 0.0000436 |
| RBL1 | -1.147 | 0.0003522 |
| ARC | -1.147 | 0.0005425 |
| ARHGAP11A | -1.144 | 0.0009582 |
| LOC280665 | -1.138 | 0.0015346 |
| TTC6 | -1.137 | 0.0004651 |
| LAMA2 | -1.135 | 0.0009643 |
| TMEM37 | -1.101 | 0.0003306 |
| SNORA36B | -1.09 | 0.0006731 |
| PPCS | -1.088 | 0.0001752 |
| CLMP | -1.084 | 0.0002022 |
| FGL1 | -1.081 | 0.0002945 |
| TLR4 | -1.076 | 0.0006201 |
| SNAR-D | -1.071 | 0.000759 |
| KLRC3 | -1.061 | 0.0006713 |
| XLOC 011616 | -1.04 | 0.0001546 |
| ZIC4 | -1.038 | 0.0006699 |
| EXOC3L2 | -1.036 | 0.000155 |
| GINS4 | -1.031 | 0.0008745 |
| ISPD | -1.027 | 0.0009247 |
| XLOC 000048 | -1.024 | 0.0003347 |
| MEGF11 | -1.016 | 0.0008533 |
| XLOC 012578 | -1.007 | 0.0003309 |
| LOC100288449 | -1.006 | 0.0005817 |
