# Supplementary material for: Prediction of novel target genes and pathways involved in bevacizumab-resistant colorectal cancer
Source: PLoS One. 2018 Jan 17;13(1):e0189582. doi: 10.1371/journal.pone.0189582 (PMC5771567; doi:10.1371/journal.pone.0189582)
Supplement: S2 Table — (DOCX) [file pone.0189582.s002.docx]

**S2Table 2: Up-regulated genes**

| **GENE SYMBOL** | **LOG2FC** | **P-VALUE** |
| --- | --- | --- |
| XLOC 001393 | 4.463 | 0.0009618 |
| CELA3B | 4.334 | 0.0005662 |
| XLOC 010456 | 3.843 | 0.0002659 |
| XLOC 005584 | 3.372 | 0.001302 |
| XLOC 001219 | 3.22 | 0.0011487 |
| MMP1 | 2.81 | 0.0008293 |
| IGFBP7 | 2.759 | 0.0011343 |
| KIRREL | 2.498 | 0.0007459 |
| FER1L6 | 2.48 | 0.0002058 |
| DACT1 | 2.431 | 0.0014192 |
| C6orf222 | 2.372 | 0.0007344 |
| LOC645638 | 2.348 | 0.0002077 |
| CDKN1A | 2.26 | 0.0000426 |
| XLOC l2 009281 | 2.202 | 0.0005678 |
| CEACAM5 | 2.186 | 0.0005058 |
| LOC645638 | 2.017 | 0.0000258 |
| ZNF462 | 2.009 | 0.0001435 |
| NR1H4 | 1.975 | 0.0001143 |
| XLOC 004917 | 1.927 | 0.0000862 |
| CHRM5 | 1.903 | 0.0010395 |
| CEACAM6 | 1.851 | 0.0001291 |
| TMEM176B | 1.824 | 0.0002705 |
| MYOM1 | 1.752 | 0.0008305 |
| AHNAK2 | 1.656 | 0.0009514 |
| RASSF10 | 1.64 | 0.000673 |
| XLOC 002650 | 1.61 | 0.0010957 |
| CTSE | 1.559 | 0.0004387 |
| SYT13 | 1.556 | 0.0002664 |
| CTSE | 1.54 | 0.0004069 |
| PDGFA | 1.485 | 0.0005813 |
| ACP5 | 1.46 | 0.0009102 |
| VNN2 | 1.419 | 0.0005386 |
| TMEM176A | 1.412 | 0.0003036 |
| LOC400084 | 1.402 | 0.0007988 |
| PBX1 | 1.383 | 0.0010895 |
| GPRIN2 | 1.366 | 0.000563 |
| LOC643406 | 1.366 | 0.0012645 |
| SQRDL | 1.354 | 0.0000306 |
| PPCS | 1.321 | 0.0009189 |
| XLOC 013838 | 1.295 | 0.0010464 |
| RTP4 | 1.254 | 0.0005349 |
| LOC100289187 | 1.246 | 0.0012665 |
| KCTD15 | 1.24 | 0.0001719 |
| FLJ44511 | 1.231 | 0.0002894 |
| TLR1 | 1.124 | 0.000104 |
| CERCAM | 1.124 | 0.0001241 |
| MTSS1 | 1.088 | 0.0005162 |
| XLOC 012515 | 1.088 | 0.0014459 |
| HEG1 | 1.087 | 0.0010535 |
| LENG9 | 1.078 | 0.0010385 |
| CDHR2 | 1.077 | 0.0005507 |
| CYP3A7 | 1.073 | 0.0015448 |
| LOC728208 | 1.042 | 0.001406 |
| DUSP5 | 1.041 | 0.0005786 |
| HOXC4 | 1.027 | 0.0007541 |
| VNN1 | 1.023 | 0.0011951 |
